# Laboratory evidence of disseminated intravascular coagulation is associated with a fatal outcome in children with cerebral malaria despite an absence of clinically evident thrombosis or bleeding

**DOI:** 10.1111/jth.13060

**Published:** 2015-08-27

**Authors:** C. A. Moxon, N. V. Chisala, R. Mzikamanda, I. MacCormick, S. Harding, C. Downey, M. Molyneux, K. B. Seydel, T. E. Taylor, R. S. Heyderman, C.‐H. Toh

**Affiliations:** ^1^ Institute of Infection and Global HealthUniversity of LiverpoolLiverpoolUK; ^2^ Malawi–Liverpool Wellcome Clinical Research ProgrammeUniversity of Malawi College of MedicineBlantyreMalawi; ^3^ University of Malawi College of MedicineBlantyreMalawi; ^4^ Institute of Aging and Chronic DiseaseUniversity of LiverpoolLiverpoolUK; ^5^ Roald Dahl Haemostasis & Thrombosis CentreRoyal Liverpool University HospitalLiverpoolUK; ^6^ Liverpool School of Tropical MedicineLiverpoolUK; ^7^ College of Osteopathic MedicineMichigan State UniversityEast LansingMIUSA; ^8^ Blantyre Malaria ProjectUniversity of Malawi College of MedicineBlantyreMalawi

**Keywords:** blood coagulation, disseminated intravascular coagulation, endothelial cell protein C receptor, fibrin, malaria, cerebral

## Abstract

**Background:**

A procoagulant state is implicated in cerebral malaria (CM) pathogenesis, but whether disseminated intravascular coagulation (DIC) is present or associated with a fatal outcome is unclear.

**Objectives:**

To determine the frequency of overt DIC, according to ISTH criteria, in children with fatal and non‐fatal CM.

**Methods/patients:**

Malawian children were recruited into a prospective cohort study in the following diagnostic groups: retinopathy‐positive CM (*n* = 140), retinopathy‐negative CM (*n* = 36), non‐malarial coma (*n* = 14), uncomplicated malaria (UM), (*n* = 91), mild non‐malarial febrile illness (*n* = 85), and healthy controls (*n* = 36). Assays in the ISTH DIC criteria were performed, and three fibrin‐related markers, i.e. protein C, antithrombin, and soluble thrombomodulin, were measured.

**Results and conclusions:**

Data enabling assignment of the presence or absence of ‘overt DIC’ were available for 98 of 140 children with retinopathy‐positive CM. Overt DIC was present in 19 (19%), and was associated with a fatal outcome (odds ratio [OR] 3.068; 95% confidence interval [CI] 1.085–8.609; *P* = 0.035]. The levels of the three fibrin‐related markers and soluble thrombomodulin were higher in CM patients than in UM patients (all *P* < 0.001). The mean fibrin degradation product level was higher in fatal CM patients (71.3 μg mL^−1^ [95% CI 49.0–93.6]) than in non‐fatal CM patients (48.0 μg mL^−1^ [95% CI 37.7–58.2]; *P* = 0.032), but, in multivariate logistic regression, thrombomodulin was the only coagulation‐related marker that was independently associated with a fatal outcome (OR 1.084 for each ng mL^−1^ increase [95% CI 1.017–1.156]; *P* = 0.014). Despite these laboratory derangements, no child in the study had clinically evident bleeding or thrombosis. An overt DIC score and high thrombomodulin levels are associated with a fatal outcome in CM, but infrequently indicate a consumptive coagulopathy.

## Introduction

Cerebral malaria (CM) is a severe complication of *Plasmodium falciparum* infection defined by unrousable coma, *P. falciparum* parasitemia, and exclusion of other causes of coma 1. CM is most frequent in children in sub‐Saharan Africa, in whom it causes approximately 600 000 cases annually and 100 000 deaths 2. The case fatality rate is 10–20% 1, and there is currently no effective adjunctive therapy 3. A number of publications have indicated a contribution to CM pathogenesis of coagulation disorders 4, 5, 6, 7, 8, 9. In the light of new treatments aimed at the coagulation–inflammation interface, including specific thrombin 10 and factor Xa inhibitors 11 and recombinant proteins 12, 13, coagulopathy has been proposed as a potential target for an adjunctive therapy to improve outcome 14, 15, 16, 17, 18. However, in non‐immune adults with CM, the relative rarity of clinically evident bleeding or decompensation of coagulation factors has been interpreted as indicating that coagulopathy is important in only a small proportion of individuals 19. Large studies that have included a variety of malaria syndromes seem to indicate that bleeding is rare in children with CM 20, but no study has provided specific data on the frequency of bleeding or thrombosis in African children with CM 21.

Several recent lines of evidence indicate that the activation of coagulation may play a role in CM pathogenesis. *In vitro*,* P. falciparum*‐infected erythrocytes (IEs) can promote the development of a procoagulant endothelial surface through induction of tissue factor 5 and by directly binding to the active site of the anticoagulant receptor endothelial protein C receptor (EPCR) 4. In postmortem samples from African children with CM, we have shown that there is marked fibrin deposition, localized to sites of IE sequestration and associated with localized loss of the anticoagulant receptors EPCR and thrombomodulin 16. Significantly raised plasma thrombin–antithrombin complex (TAT) levels in patients with CM imply that there is sufficient activation of coagulation for this to be detected in the systemic circulation. The fact that levels of circulating activated protein C‐to‐prothrombin fragment ratio (F_1+2_) are similar in CM and uncomplicated malaria (UM) suggests that coagulation is generally compensated in CM patients 16. These data provide clear evidence of a localized coagulopathy in the brain in CM, but it is unclear whether the activation of coagulation detected in plasma simply represents the downstream detection of a purely cerebral event or whether, in some individuals, IE‐induced loss of anticoagulant function may cause a systemic disturbance of coagulation and lead to disseminated intravascular coagulation (DIC).

To enable standardization between studies on the definition of DIC, the ISTH has devised a scoring system based on the prothrombin time (PT), the platelet count, and the circulating levels of fibrinogen and a soluble fibrin marker. We therefore measured these and other coagulation indices in several groups of Malawian children: those who met the case definition of CM; encephalopathic aparasitemic patients; children with UM; children with aparasitemic uncomplicated febrile illness; and healthy controls (HCs). The objective of this study was to examine the association between the overt DIC score and its components and mortality.

## Materials and methods

### Study population

Children aged 6 months to 12 years were recruited into a prospective cohort study at Queen Elizabeth Central Hospital, Blantyre, Malawi between January 2008 and July 2011, and constitute the same cohort of patients as in previously published data 16, 22. Some of the data presented here (PT, platelet counts, TAT levels and soluble thrombomodulin [sTM] levels from the non‐comatose patients only) were included in previously published reports 16, 22, but are presented here to enable calculation of the DIC score and to compare them with other factors analyzed for the first time. Six patient groups were prospectively defined: retinopathy‐positive CM, retinopathy‐negative CM, aparasitemic coma, UM, mild aparasitemic febrile illness, and HCs. In high‐transmission areas such as Malawi, where a significant proportion of apparently well children are parasitemic, the diagnosis of CM according to World Health Organization (WHO) criteria 23 leads to misclassification in ~ 25% of cases 1. In a parasitemic comatose child, it is unclear whether the parasitemia has caused the coma or whether another etiology – infectious or non‐infectious – has precipitated coma in a child who already had asymptomatic parasitemia. Classification of CM patients as retinopathy‐positive or retinopathy‐negative, as assessed by an ophthalmologist using dilated ophthalmoscopy 24, has emerged as an important method for refining the diagnosis of CM. The presence or absence of retinal whitening, vessel changes and/or microhemorrhages is associated with sequestration of IEs in cerebral vessels 25, and distinguishes definitive retinopathy‐positive CM from retinopathy‐negative parasitemic encephalopathies, in which another etiology is indicated as a cause of coma 1. Non‐malarial coma was defined as a Blantyre coma score of ≤ 2 in the absence of parasitemia and in the absence of overt meningitis (i.e. cloudy cerebrospinal fluid). The group includes children with both infectious and non‐infectious etiologies of coma, but the etiology of coma is often not identified 26. UM and mild aparasitemic febrile illness patients had acute febrile illness without signs of organ compromise 20, and did not require hospital admission. HCs were children attending for elective surgery who were otherwise well, who did not have a history of systemic illness, and who were not receiving regular medication. Patients were observed until discharge or death. The study was approved by ethical boards at the College of Medicine in Malawi (no. P.02/10/860) and the Liverpool School of Tropical Medicine in the UK (no. 09.74). Informed consent was obtained from the parents or guardians of all of the children enrolled.

### Global and molecular markers of coagulation activation

Blood was obtained by venepuncture at a single time point on admission to hospital, and citrated plasma was prepared as described previously 27. Samples were frozen immediately, and stored at − 80 °C. Antithrombin activity, protein C activity, the fibrinogen level (Claus method) and the PT were determined on an MDA180 analyzer (Stago, Düsseldorf, France). Levels of fibrin monomers, d‐dimers, fibrin and fibrin degradation products (FDPs) were determined on an STA Compact CT analyzer (Stago). F_1+2_, TAT and sTM levels were determined with commercial ELISA kits (Enzygnost TAT micro [Dade Behring, Marburg, Germany]; sTM DY3947 [R&D, Minneapolis, MN, USA]). DIC was defined according to ISTH criteria 28. Normal values relevant to the study population were defined as those in the Malawian pediatric HCs.

### Statistical methods

Statistical analysis was performed with stata (version 11; Statacorp, College Station, TX, USA) and prism (version 5.0; GraphPad, La Jolla, CA, USA) software. Continuous variables were generally assumed to have a normal or a log normal distribution, depending on their level of skewness. Differences between groups for these variables were compared by the use of either a *t*‐test or anova; for the latter, the Tukey honestly significant difference test was used to adjust for multiple comparisons. The relationship between fatal outcome and baseline clinical and laboratory variables was assessed by the use of odds ratios (ORs) and a logit model, with stata software. The variables assessed were those that have previously been shown to be associated with severe disease, i.e. admission glucose level, hemoglobin level, histidine‐rich protein‐2 (HRP2) level, parasite count, and lactate level, and all of the coagulation indices assessed in this study (PT, fibrinogen level, fibrin monomer level, FDP level, d‐dimer level, antithrombin activity, protein C activity, and thrombomodulin level). The OR indicates the estimated increase in the log odds of a fatal outcome per unit increase (e.g. thrombomodulin, each ng mL^−1^; lactate, each mmol L^−1^) for each continuous variable. To adjust for age and sex, these variables were included as independent variables in the model. Missing data were handled by listwise deletion. All tests were two‐tailed with a conventional 5% *α*‐level.

## Results

The numbers of patients included in each of the groups were as follows: retinopathy‐positive CM, *n* = 140; retinopathy‐negative CM, *n* = 36; non‐malarial coma, *n* = 14; UM, *n* = 91; mild non‐malarial febrile illness (MF), *n* = 85; and HCs, *n* = 36. The case fatality rate did not differ significantly between children with retinopathy‐positive CM (16.4%) and those with retinopathy‐negative CM (13.9%) or non‐malarial coma (13%) (*P* = 0.8; the case definitions and significance of these three encephalopathic groups are detailed under ‘Study population’ in Materials and methods). Outcome data were complete for all hospitalized patients. Other parameters are compared in Table 1.

**Table 1:** Clinical characteristics of the children

	Healthy controls (*n* = 36)	Mild febrile illness (*n* = 85)	Uncomplicated malaria (*n* = 91)	Non‐malarial coma (*n* = 14)	Retinopathy‐negative CM (*n* = 36)	Retinopathy‐positive CM (*n* = 140)
Age (months), median (IQR)	57 (39–94)	39 (24–62)*	65 (40–84)	56 (31–64)	45 (28–52)	45 (33–62)
Female sex, *n* (%)	13 (36)	41 (44)	53 (55)	8 (57)	17 (52)	76 (56.3)
HIV‐positive, *n* (%)	0 (0)	0 (0)	1 (3)	0 (0)	4 (9.5)	11 (8.09)
Findings at presentation, median (IQR)						
Axillary temperature (°C)	36.8 (36.2–36.8)	38.4 (37.9–38.7)***	38.4 (37.9–39.0)***	38.3 (37.5–39.5)***	39.1 (38.1–39.8)***	38.8 (38.0–39.5)***
Pulse rate (beats min^−1^)	108 (102–121)	137 (115–153)*	140 (120–151)*	150 (103–162)**	154 (136–164)***	152 (136–172)***
Systolic blood pressure (mmHg)	107.5 (97–117)	118 (106–125)	113 (105–121)	98 (90–107)	100 (90–105)*	98 (90–109)*
Respiratory rate (breaths min^−1^)	28 (24–35)	32 (26–34)	28 (26–32)	45 (30–60)***	42 (38–53)***	44 (37–52)***
Blood glucose (mmol L^−1^)	4.9 (4.5–5.5)	5.2 (4.6–5.7)	5.7 (4.9–6.6)	5.8 (4.3–8.8)*	6.4 (5.3–8.9)***	6.3 (5.2–7.8)***
Blood lactate (mmol L^−1^)	1.8 (1.6–2.1)	1.7 (1.2–2.2)*	2.2 (1.9–2.9)	3.3 (2.1–5.0)**	3.2 (2.4–7.4)***	6.2 (3.3–10.3)***
Hb (g L^−1^)	102 (90–108)	110 (98–118)**	94 (81–108)	90 (74–100)	83 (72–99)*	64 (50–80)***
Platelets (× 10^9^ L^−1^)	435 (309–476)	327 (237–387)**	112 (75–144)***	263 (145–512)*	128 (60–219)***	60 (32–96)***
Parasitemia (parasites × 10^3^ μL^−1^)	0	0	22 (1.6–173)***	0	56 (13–245)***	85 (10–278)***

CM, cerebral malaria; Hb, hemoglobin; IQR, interquartile range. HIV was tested for by the use of rapid testing (Determine; Inverness Medical, Inverness, UK). Missing data (missing at random) were as follows. Age: one retinopathy‐positive CM case. Female sex: one retinopathy‐positive CM case. Hb: four mild febrile illness cases, and three retinopathy‐positive CM cases. Platelets: nine mild febrile illness cases, six uncomplicated malaria cases, one non‐malarial coma case, one retinopathy‐negative CM case, and 11 retinopathy‐positive CM cases. Parasitemia: 38 uncomplicated malaria cases. If not stated above, data were complete. For each variable, differences between healthy controls and other patient groups were examined with a Mann–Whitney *U* test (continuous variables) or Fisher's exact test (categorical variables). *P < 0.05; **P < 0.01; ***P < 0.001.

### Evidence of fibrin generation

**Figure 1:**
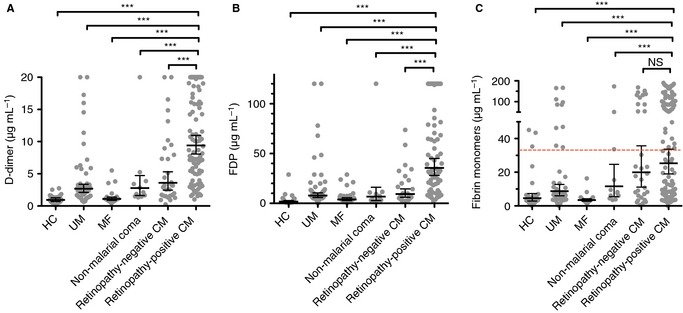
Increased plasma markers of intravascular fibrin generation in cerebral malaria (CM). (A) Plasma d‐dimer levels measured on admission/presentation in children with retinopathy‐positive CM (*n* = 102), retinopathy‐negative CM (*n* = 31), non‐malarial coma (*n* = 11), uncomplicated malaria (UM) (*n* = 55), and mild non‐malarial febrile illness (MF), (*n* = 28), and in healthy controls (HCs) (*n* = 27). (B) Plasma fibrin degradation product (FDP) levels in children with retinopathy‐positive CM (*n* = 74), retinopathy‐negative CM (*n* = 26), non‐malarial coma (*n* = 10), MF (*n* = 34), and UM (*n* = 64), and in HCs (*n* = 24). (C) Plasma fibrin monomers in children with retinopathy‐positive CM (*n* = 114), retinopathy‐negative CM (*n* = 32), non‐malarial coma (*n* = 12), MF (*n* = 25), and UM (*n* = 51), and in HCs (*n* = 28). The dashed horizontal line across columns in (C) indicates two standard deviations from the mean in HCs, the threshold for this index in the overt disseminated intravascular coagulation score. The solid horizontal lines in (A)–(C) indicate geometric means, and the bars indicate 95% confidence intervals. Significance was determined by anova with the Tukey honestly significant difference test on log‐transformed data to adjust for multiple comparisons. ***P < 0.001. NS, not significant.

The plasma levels of several different markers of fibrin breakdown – d‐dimers, FDPs, and soluble fibrin monomers (Fig. 1) – all indicated greater fibrin generation and breakdown in retinopathy‐positive CM patients than in the non‐comatose comparator groups (HC, MF, and UM; all *P* < 0.001). There was evidence of moderately increased fibrin production in children with non‐malarial coma and retinopathy‐negative CM. Significant differences (Fig. 1A,B) between children with retinopathy‐positive CM and these other comatose clinical groups indicated that fibrin generation was particularly increased in retinopathy‐positive CM patients. Among retinopathy‐positive patients, the geometric mean FDP level was significantly higher in children who went on to die (71.3 μg mL^−1^; 95% confidence interval [CI] 49.0–93.6) than in those who survived (48.0 μg mL^−1^; 95% CI 37.7–58.2; *P* = 0.032), but the mean d‐dimer and fibrin monomer levels, although higher in survivors, were not significantly so (*P* = 0.25 and *P* = 0.44, respectively). Taken together, these data indicate that fibrin production is associated with the diagnosis of retinopathy‐positive CM, and that patients with high levels of fibrin generation are more likely to die.

### Frequency of overt DIC in CM and its association with a fatal outcome

**Figure 2:**
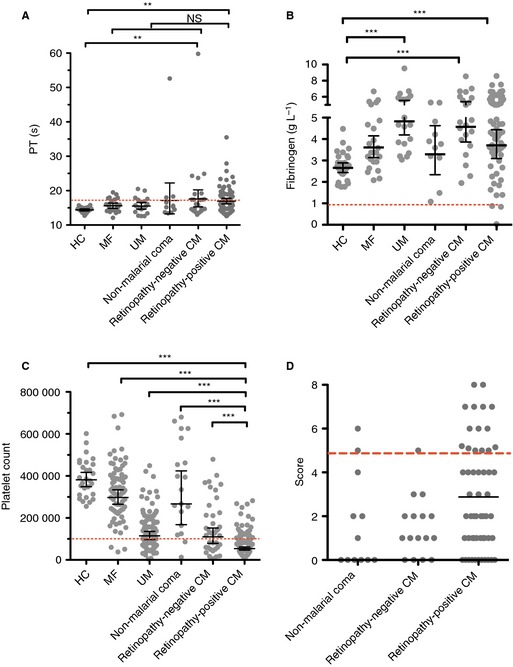
ISTH score for overt disseminated intravascular coagulation (DIC) and its components in cerebral malaria (CM) patients and comatose and non‐comatose controls. (A, B) Prothrombin time (PT) (A) and fibrinogen (B) in children with retinopathy‐positive CM (*n* = 70), retinopathy‐negative CM (*n* = 23), non‐malarial coma (*n* = 11), uncomplicated malaria (UM) (*n* = 21), and mild non‐malarial febrile illness (MF) (*n* = 24), and in healthy controls (HCs) (*n* = 30). The red dashed lines in (A) and (B) indicate a PT prolonged by 3 s over the geometric mean of the HCs (≥ 17.4 s) and a fibrinogen level of > 1 g L^−1^, the threshold for these indices in the overt DIC score. (C) Platelet count (×10^6^ L^−1^) in children with retinopathy‐positive CM (*n* = 138), retinopathy‐negative CM (*n* = 39), non‐malarial coma (*n* = 19), UM (*n* = 88), and MF (*n* = 77), and in HCs (*n* = 28). The red dashed line indicates a platelet count of 100 000 × 10^6^ L^−1^. (D) ISTH DIC scores in children with retinopathy‐positive CM (*n* = 55), retinopathy‐negative CM (*n* = 16), and non‐malarial coma (*n* = 12). For 43 retinopathy‐positive CM patients, 10 retinopathy‐negative CM patients, and two non‐malarial coma patients, data were missing for one or two components, and so a score is not shown, but either the score was already ≥ 5 or it would not reach ≥ 5 regardless of the missing data, and so the presence or absence of overt DIC could still be assigned with certainty. The red dashed line indicates a score of ≥ 5, the threshold for overt DIC. Horizontal lines indicate geometric means, and bars indicate 95% confidence intervals. Significance was determined by anova with the Tukey honestly significant difference test on log‐transformed data to adjust for multiple comparisons. **P < 0.01, ***P < 0.001. The data in (A) and (C) have been published previously 16, but are presented again here for comparison with other coagulation parameters and to enable calculation of the ISTH DIC score. NS, not significant.

The ISTH scoring system for overt DIC makes use of the PT, the platelet count, and fibrinogen levels, in addition to a soluble fibrin marker, to calculate a combined coagulation score 29. The mean PT was significantly prolonged in both retinopathy‐positive CM and retinopathy‐negative CM patients as compared with HCs (Fig. 2A; both *P* < 0.01), but was not significantly different between retinopathy‐positive CM and retinopathy‐negative CM patients, or between retinopathy‐positive CM patients and the other clinical groups (Fig. 2A). Specifically for children with retinopathy‐positive CM, the PT was similar between children with a fatal outcome (geometric mean, 18.3 s; 95% CI 15.9–22.4 s) and children who survived (16.6 s; 95% CI 15.9–17.4 s; *P* = 0.06). Consistent with previously published data 30, the platelet count was markedly reduced in UM, retinopathy‐positive CM and retinopathy‐negative CM patients, and was lower in retinopathy‐positive CM patients than in all of the other clinical groups (Fig. 2C; all *P* < 0.001). However, as previously shown 30, in retinopathy‐positive CM patients, the platelet count was not significantly associated with a fatal outcome (fatal, 60 262 platelets dL^−1^, 95% CI 42 820–77 705; non‐fatal, 67 069 platelets dL^−1^, 95% CI 60 275–73 864; *P* = 0.43). The geometric mean fibrinogen levels in retinopathy‐positive CM patients were significantly higher than those in HCs, although there was a wide spread of data (Fig. 2B). All except three retinopathy‐positive CM patients had fibrinogen levels of > 1 g L^−1^, the defined lower cut‐off taken to indicate consumptive coagulopathy in the ISTH scoring system, and no patients reached this cut‐off in any of the comparator groups. In the subgroup of retinopathy‐positive children, there was no association between fibrinogen levels and fatal outcome (fatal, mean of 3.6 g L^−1^, 95% CI 2.2–5.0 g L^−1^; non‐fatal, mean of 4.4 g L^−1^, 95% CI 4.0–4.8 g L^−1^; *P* = 0.18). We used fibrin monomers rather than d‐dimer or FDPs in the calculation of the DIC score, as there is evidence that it is a more specific marker of coagulation activation and fibrin generation 31, 32; however, the fibrin marker used had only a minor effect on the assignment of DIC score (data not shown).

A complete set of parameters were available for 55 retinopathy‐positive CM patients, 16 retinopathy‐negative CM patients, and 12 non‐malarial coma patients (Fig. 2D). In additional patients, although one or two parameters were missing, either the score was already ≥ 5 or would not reach ≥ 5 irrespective of the value of the missing parameters, and so the presence or absence of overt DIC could still be assigned with certainty. A score indicative of overt DIC (≥ 5) was present in 19 of 98 (19%) retinopathy‐positive CM patients, two of 26 (8%) retinopathy‐negative CM patients, and two of 14 (14%) non‐malarial coma patients. In the subgroup of retinopathy‐positive CM patients, 10 (32%) of the 31 patients with an overt DIC score died (OR 3.068; 95% CI 1.085–8.609; *P* = 0.035), as compared with nine (13.4%) of the 67 patients who did not have overt DIC, and eight (19%) of the 42 patients for whom we did not have all of the data needed to calculate a DIC score. Among retinopathy‐negative CM patients, only two patients had overt DIC, both of whom survived. Five (21%) of 17 patients who did not have overt DIC died, as compared with two (10%) of the 20 patients for whom we did not have all of the data needed to calculate a DIC score.

Several case reports have described an association between quinine therapy and DIC 33. Given that the majority of our patients had received quinine therapy prior to blood sampling, either in a peripheral health center or immediately on arrival at hospital, we examined whether overt DIC was associated with prior quinine therapy. Data on prior quinine therapy were available for 63 patients: 16 who had overt DIC, and 49 who did not. Eleven of the 16 patients (69%) who had overt DIC received quinine prior to blood being taken, as compared with 43 of the 47 patients (91%) who did not have overt DIC. Prior quinine treatment was therefore associated with protection from rather than an increased risk of overt DIC (OR 0.205; 95% CI 0.035–1.160; *P* = 0.039).

These data indicate that there is an association between a prothrombotic state and the diagnosis of retinopathy‐positive CM, and that, within this group, a prothrombotic state is associated with an adverse outcome. Clinically apparent bleeding or thrombosis was not noted in any patient in this study, including the 19 CM patients with a positive overt DIC score. To ensure that we had not, through bias in recruitment of blood samples, missed significant coagulopathies associated with CM in the population of Malawian children presenting to our unit, we conducted a notes review of all 104 patients admitted with CM in 2008, and did not identify clinically evident bleeding or thrombosis in any of them.

### Anticoagulant factors in CM

**Figure 3:**
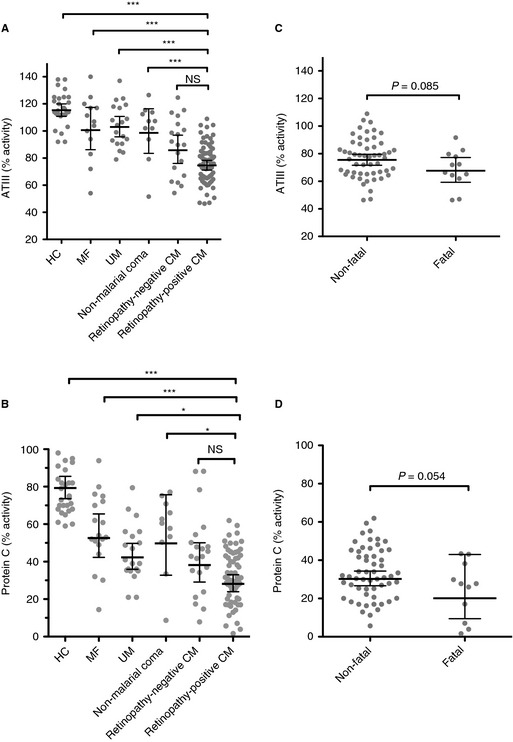
Decreased anticoagulant activity in cerebral malaria (CM) and association with outcome. (A) Antithrombin activity measured at admission in children with retinopathy‐positive CM (*n* = 68), retinopathy‐negative CM (*n* = 19), non‐malarial coma (*n* = 11), uncomplicated malaria (UM) (*n* = 19), and mild non‐malarial febrile illness (MF) (*n* = 13), and in healthy controls (HCs) (*n* = 29). (B) Protein C activity in children with retinopathy‐positive CM (*n* = 68), retinopathy‐negative CM (*n* = 21), non‐malarial coma (*n* = 11), UM (*n* = 20), and MF (*n* = 18), and in HCs (*n* = 30). (C) Antithrombin activity at admission in patients with retinopathy‐positive CM grouped according to outcome: those who eventually died (fatal, *n* = 12) and those who survived (non‐fatal, *n* = 54). (D) Protein C activity in fatal (*n* = 12) and non‐fatal cases (*n* = 56) of retinopathy‐positive CM. Horizontal lines indicate means, and bars indicate 95% confidence intervals. In (A) and (B), significance was determined by anova with the Tukey honestly significant difference test to adjust for multiple comparisons. In (C) and (D), significance was determined with a two‐tailed *t*‐test. *P < 0.05, ***P < 0.001. ATIII, antithrombin; NS, not significant.

Both antithrombin activity (Fig. 3A) and protein C activity (Fig. 3B) were decreased in retinopathy‐positive CM patients as compared with non‐comatose controls (HC, MF, and UM; all *P* < 0.001) and non‐malarial coma patients (*P* < 0.001). In CM patients, protein C activity was lower than antithrombin activity, but the percentage differences between HCs and retinopathy‐positive CM or retinopathy‐negative CM patients were similar for both molecular markers (Fig. 3A,B). In the subgroup of retinopathy‐positive CM children, antithrombin activity and protein C activity were not significantly different in fatal and non‐fatal cases (Fig. 3C,D).

### sTM levels in CM

**Figure 4:**
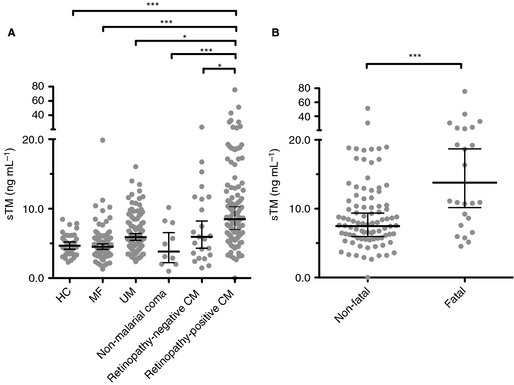
Increased plasma soluble thrombomodulin (sTM) levels in children with cerebral malaria (CM) and association with fatal outcome. (A) Plasma sTM levels measured at admission in children with retinopathy‐positive CM (*n* = 115), retinopathy‐negative CM (*n* = 23), non‐malarial coma (*n* = 10), uncomplicated malaria (UM) (*n* = 103), and mild non‐malarial febrile illness (MF) (*n* = 95), and in healthy controls (HCs) (*n* = 36). (B) Admission sTM levels in fatal (*n* = 24) and non‐fatal (*n* = 91) cases. Horizontal lines indicate means, and bars indicate 95% confidence intervals. Significance in (A) was determined by anova with the Tukey honestly significant difference test to adjust for multiple comparisons. Significance in (B) was determined with a two tailed *t*‐test. *P < 0.05, ***P < 0.001. The data from the HCs and from the MF and UM patients have been published previously 22, but are presented again here for comparison with new data from non‐malarial comatose, retinopathy‐negative CM and retinopathy‐positive CM patients, and in the context of outcome.

Plasma levels of sTM constitute an established marker of endothelial damage in DIC 34, and we have shown that thrombomodulin is lost at sites of IE sequestration and fibrin deposition in CM 16. The geometric mean plasma sTM level was significantly higher in retinopathy‐positive CM patients than in all of the other clinical groups (Fig. 4A). Among retinopathy‐positive CM patients, geometric mean sTM levels were higher in patients who died (14.5 ng mL^−1^; 95% CI 9.5–22.1) than in those who survived (8.2 ng mL^−1^; 95% CI 6.6–10.1; *P* < 0.001; Fig. 4B).

### Risk factors associated with a fatal outcome in CM patients

A logistic regression model was generated, adjusting for age and sex, with outcome (fatal or non‐fatal) as the dependent variable, and factors associated with severe malaria and coagulation indices as independent variables (plasma lactate, hemoglobin, blood glucose, parasite count, and HRP2). The results of univariate analysis are shown in Table 2. Lactate, glucose, thrombomodulin, TATs, FDPs and overt DIC were significantly associated with mortality on univariate regression. When a multivariate logistic regression model was employed, only sTM (OR 1.084; 95% CI 1.017–1.156; *P* = 0.014), blood glucose (OR 1.218; 95% CI 1.051–1.412; *P* = 0.009 and plasma lactate (OR 1.204; 95% CI 1.049–1.382; *P* = 0.008) were independent predictors of mortality (Table 3). As a fibrin marker (fibrin monomers) is already used to determine overt DIC, to avoid collinearity, only FDPs or overt DIC were used in any given model. Nonetheless, although both were significantly associated with mortality on univariate analysis, neither overt DIC nor FDP levels were independently predictive of fatal outcome when glucose, thrombomodulin, lactate, age and sex were included in the model.

**Table 2:** Univariate analysis of association of covariates with a fatal outcome

Covariate	*N*	OR	95% CI	*P*
Age (months)	139	0.983	0.964–1.002	0.083
Sex (male/female)	139	0.862	0.365–2.029	0.733
Prothrombin time (s)	67	1.126	0.970–1.306	0.118
Fibrin monomers (μg mL^−1^)	114	1.003	0.996–1.010	0.422
Fibrin degradation products (μg mL^−1^)	74	1.013	1.001–1.025	0.035
d‐dimers (μg mL^−1^)	102	1.039	0.967–1.117	0.292
Fibrinogen (g L^−1^)	67	0.997	0.993–1.001	0.167
Thrombin–antithrombin complexes (μg L^−1^)	74	1.016	1.003–1.027	0.010
Antithrombin activity (%)	66	0.969	0.927–1.014	0.149
Protein C activity (%)	65	1.000	0.967–1.036	0.967
Platelets (× 10^6^ L^−1^)	129	1.000	1.000–1.000	0.925
Overt DIC (yes/no)	98	3.068	1.085–8.609	0.035
DIC score	55	1.384	1.041–1.841	0.025
Thrombomodulin (ng mL^−1^)	115	1.091	1.029–1.158	0.004
Lactate (mmol L^−1^)	140	1.174	1.061–1.300	0.002
Histidine‐rich protein‐2 (ng mL^−1^)	51	1.001	0.999–1.003	0.496
Parasitemia (parasites μL^−1^)	136	1.000	1.000–1.000	0.835
Hemoglobin (g dL^−1^)	137	0.947	0.761–1.178	0.625
Blood glucose (mmol L^−1^)	140	1.138	1.000–1.295	0.049

CI, confidence interval; DIC, disseminated intravascular coagulation; OR, odds ratio. The OR indicates the estimated increase in the log odds of a fatal outcome per unit increase (e.g. thrombomodulin, each ng mL^−1^; lactate, each mmol L^−1^) for each continuous variable.

**Table 3:** Multivariate logistic regression analysis of mortality outcome with age, sex and thrombomodulin, glucose and lactate levels as covariates

	*N*	OR	95% CI	*P*
Age	114	0.986	0.951–1.008	0.160
Sex	114	1.170	0.746–17.056	0.111
Thrombomodulin	114	1.084	1.017–1.156	0.014
Plasma lactate	114	1.204	1.049–1.382	0.008
Blood glucose	114	1.218	1.051–1.412	0.009

CI, confidence interval; OR, odds ratio. The OR indicates the estimated increase in the log odds of a fatal outcome per unit increase (e.g. thrombomodulin, each ng mL^−1^; lactate, each mmol L^−1^) for each continuous variable.

### Effect of HIV on DIC score and coagulation indices

Eleven children in the retinopathy‐positive group were HIV‐positive. WHO clinical staging data 35 were available for six patients, all of whom were clinical stage 1. We compared the different coagulation parameters in the HIV‐positive and HIV‐negative children, and found no evidence of an effect of HIV on any parameter or on the DIC score (Table S1).

### No association between plasma coagulation makers and retinal hemorrhages in CM

Although the vasculature of the brain is inaccessible during life, examination of the retinal neurovasculature by funduscopy provides ‘a window into the brain’, with a high correlation between premortem retinal changes *in vivo* and postmortem vasculature changes in the brain 36. To assess whether intravascular coagulation in the neurovasculature is associated with a procoagulant state, we assessed the association of fibrin markers, activation of coagulation and DIC score with retinal hemorrhages. Among retinopathy‐positive CM patients, we had data on both DIC score and retinal examination for 59 patients. A positive score for overt DIC was not associated with the presence of retinal hemorrhages (OR 0.59; 95% CI 0.21–1.69; *P* = 0.33). On univariate analysis, none of the fibrin markers (fibrin monomers, FDPs, or d‐dimers), coagulation measurements (PT, TAT, antithrombin III, protein C, and fibrinogen), platelet count or DIC score was significantly associated with retinal hemorrhages (*P* > 0.1).

## Discussion

We have systematically investigated the presence of DIC in African children with CM by using validated international criteria. Approximately one‐fifth of children for whom we had data to calculate the DIC score met the ISTH criteria for overt DIC, despite none of these patients having clinically evident bleeding or fulminant thrombosis.

Previously, in brain tissue from children with fatal CM, we demonstrated intravascular fibrin deposition specific to retinopathy‐positive CM, occurring at sites of sequestration of IEs in association with loss of endothelial protein C and thrombomodulin 16. The increased levels of fibrin markers shown in the current study demonstrate that fibrin formation occurs *in vivo*, and provide evidence for the thrombosis observed postmortem being not merely a perimorbid event, but being representative of a process that is occurring when children with CM present to hospital. Retinopathy‐positive CM patients had higher levels of fibrin markers and were more likely to have overt DIC than retinopathy‐negative CM or aparasitemic comatose patients – both groups of sick patients in coma with similar case fatality rates. Furthermore, retinopathy‐positive CM patients who had laboratory‐defined overt DIC at presentation were three times more likely to die than those who did not. Together with previous studies 5, 16, 37, these findings strongly indicate the the involvement of a procoagulant state and intravascular coagulation in CM pathogenesis.

However, despite a positive overt DIC score in 19 of 98 retinopathy‐positive CM patients and significantly increased fibrin generation, none of these patients had clinically apparent bleeding or major thrombotic episodes. Although bleeding is recognized in African children with CM, these and previous data 20 indicate that it is uncommon. Although there are case reports of fulminant thrombosis, such as purpura or limb ischemia, in non‐immune adults with severe malaria 38, we have not identified any such reports in children with malaria. If fulminant thrombosis does occur in African children with CM, it is very rare. This is in contrast to other systemic infections that activate coagulation, such as bacterial sepsis, in which bleeding and fulminant thrombosis are more common (serious bleeding in 7% in a recent pediatric sepsis trial 39 and purpura in 30%; limb ischemia resulting from thrombosis is less common but well described). It is also noteworthy that only three patients had low circulating fibrinogen levels, indicating that fibrinogen consumption rarely outstrips production, and hence that, systemically, coagulation remains compensated. We suggest that, in Malawian children with CM, these observations are in keeping with intravascular coagulation that is restricted to the microvasculature, at sites of IE sequestration, and particularly in the vulnerable vascular bed of the brain. The markedly raised sTM levels, which are specific to retinopathy‐positive CM, and the association between thrombomodulin levels and fatal outcome – more strongly than, and independently of, other coagulation factors – further support the role of the protein C pathway in the etiology of this intravascular coagulation in CM 4, 16, 40. Thrombomodulin, which is a key receptor in the protein C pathway, is predominantly expressed in the microvasculature, and we have previously shown its loss – putatively by receptor shedding – in association with fibrin deposition at sites of sequestration 16. Whether or not this coagulopathy is as localized in adults with CM remains to be determined.

The lack of an association between retinal hemorrhages and coagulation indices may be another indicator that elevated coagulation indices constitute a downstream and insensitive indicator of intravascular coagulation in the neurovasculature. Another potentially overlapping explanation for a lack of association between hemorrhages and coagulation indices is that hemorrhages constitute an insensitive indicator of thrombosis. In support of this, previously in postmortem brain samples, we detected fibrin in 64% of vessels in fatal CM cases, whereas we detected hemorrhage in only 2.4% of vessels 16. Whereas fibrin deposition occurred in all individuals examined, hemorrhages were present in only ~ 75% 1, 16, 41. In both retinal and cerebral tissue, it remains to be determined why hemorrhages develop in certain vessels and in some, but not all, children with CM.

The strengths of our study include the strict criteria applied in the diagnosis of CM, which was further refined by the use of retinal examination. In addition, we had well‐stratified comparator groups within the local population, with both comatose and non‐comatose cohorts, and we established normal values from healthy Malawi children. Even with a large, carefully controlled study of this kind, there will be limitations. First, undertaking the study in a pediatric population limited the volume of blood that could be collected, and this meant that we were not able to measure all factors in all patients. This limited the numbers of cases for analysis for some factors. Blood was taken at only one time point, representing a snapshot in the disease process, with the possibility that different patients are captured at different points in the course of illness. This may be particularly important when an association with mortality is examined, as we may have failed to detect an association in patients presenting early as opposed to those presenting late in their illness.

The significant intravascular fibrin production and raised thrombomodulin levels demonstrated in this study, specific to children with retinopathy‐positive CM, add to our previous data indicating a role of microvascular thrombosis, in association with disruption of the protein C pathway, in the pathogenesis of the disease. The findings provide further evidence to suggest that the protein C receptor loss and fibrin deposition in the cerebral microvasculature observed postmortem is already occurring at the time when children present to hospital. We show significant laboratory evidence that derangement of coagulation occurs in CM, and that the derangement is greatest in those patients who go on to die. In contrast to other conditions that cause a procoagulant state and lead to positive overt DIC scores, this derangement rarely leads to bleeding, fulminant thrombosis, or consumptive coagulopathy. The use as adjunctive therapy in CM of agents that prevent thrombin or fibrin generation warrants further investigation.

## Addendum

C. A. Moxon, R. S. Heyderman, and C‐H. Toh had the idea for this study. T. E. Taylor, M. E. Molyneux, and K. B. Seydel coordinated the overarching malaria pathogenesis study. N. V. Chisala, C. Downey, and C. A. Moxon performed the coagulation assays. I. MacCormick and S. Harding collated the fundoscopy data. C. A. Moxon analyzed the data and prepared the first draft of the manuscript, with input from other authors. All authors approved the final manuscript.

## Disclosure of Conflict of Interests

The authors state that they have no conflict of interest.

## Supporting information


**Table S1.** Comparison of coagulation indices between HIV‐positive and HIV‐negative children with retinopathy‐positive cerebral malaria.
