# Supplementary material for: Laboratory evidence of disseminated intravascular coagulation is associated with a fatal outcome in children with cerebral malaria despite an absence of clinically evident thrombosis or bleeding
Source: J Thromb Haemost. 2015 Aug 27;13(9):1653–64. doi: 10.1111/jth.13060 (PMC4605993; doi:10.1111/jth.13060)
Supplement: Supplementary file 1 — Table S1. Comparison of coagulation indices between HIV‐positive and HIV‐negative children with retinopathy‐positive cerebral malaria. [file JTH-13-1653-s001.docx]

|  | HIV negative | | | HIV positive | | | P value |
| --- | --- | --- | --- | --- | --- | --- | --- |
|  | N | Mean | 95% CI | N | Mean | 95% CI |  |
| Prothrombin time | 48 | 16.8 | 15.9 - 17.6 | 8 | 16.46 | 14.5 - 18.5 | 0.39 |
| Fibrin monomers | 80 | 53.9 | 40.8 - 66.9 | 10 | 70.71 | 18.6 - 122. 9 | 0.8 |
| Fibrin degredation products | 50 | 48.9 | 37.9 - 59.9 | 6 | 27.30 | 6.2 - 48.4 | 0.09 |
| D-dimers | 71 | 11.8 | 10.2 - 13.4 | 8 | 11.55 | 5.0 - 18.1 | 0.46 |
| Fibrinogen | 48 | 4.44 | 3.94 - 4.95 | 8 | 4.39 | 3.4 - 5.4 | 0.08 |
| Thrombin-anti-Thrombin complexes | 45 | 37.9 | 25.8 - 50.0 | 8 | 23.33 | 7.05 - 39.6 | 0.16 |
| Thrombomodulin | 48 | 11.1 | 8.78 - 13.5 | 8 | 9.68 | 3.39 - 16.0 | 0.32 |
| Antithrombin activity | 43 | 78.5 | 73.7 - 83.3 | 7 | 74.17 | 60.9 - 87.5 | 0.25 |
| Protein C activity | 42 | 34.2 | 30.0 - 38.4 | 6 | 28.30 | 15.2 - 41.4 | 0.16 |
| DIC score | 35 | 2.34 | 1.58 - 3.11 | 5 | 3.00 | 0 - 6.04 | 0.73 |

**Supplemental Table.** Comparison of coagulation indices between HIV positive and HIV negative children with retinopathy positive cerebral malaria.
